# Unveiling the myxofibrosarcoma tumor microenvironment: implications for immunotherapy

**DOI:** 10.3389/fimmu.2025.1717541

**Published:** 2026-01-05

**Authors:** Cecilia Profumo, Massimiliano Grassi, Valentina Rigo, Matteo Mascherini, Paola Monti, Giorgia Arcovito, Giorgia Anselmi, Laura Damele, Giorgia Timon, Danila Comandini, Michela Croce

**Affiliations:** 1 UOC Bioterapie, IRCCS Ospedale Policlinico San Martino, Genova, Italy; 2 Dipartimento di Medicina Sperimentale (DiMeS), Università di Genova, Genova, Italy; 3 Department of Internal Medicine and Medical Specialties (DIMI), University of Genova, Genova, Italy; 4 IRCCS Humanitas Research Hospital, Medical Oncology and Hematology Unit, Humanitas Cancer Center, Milan, Italy; 5 UO Clinica Chirurgica 1, IRCCS Ospedale Policlinico San Martino, Genova, Italy; 6 UO Neuro-oncologia e Mutagenesi, IRCCS Ospedale Policlinico San Martino, Genova, Italy; 7 Anatomia Patologica Ospedaliera, IRCCS Ospedale Policlinico San Martino, Genova, Italy; 8 CYMACS Lab, Cytofluorimetry and Mass-cytometry Core Service, IRCCS Ospedale Policlinico San Martino, Genova, Italy; 9 Radioterapia Oncologica, IRCCS Ospedale Policlinico San Martino, Genova, Italy; 10 Oncologia Medica, IRCCS Ospedale Policlinico San Martino, Genova, Italy

**Keywords:** myxofibrosarcoma, tumor microenvironment, biomarkers, immunotherapy, immune checkpoint inhibitors

## Abstract

Myxofibrosarcoma (MFS) is a rare and aggressive soft tissue sarcoma characterized by high genomic instability, resulting in high local recurrence rates and limited effective therapeutic options in advanced stages. Recent progress in cancer immunology research has encouraged investigation into the Tumor Microenvironment (TME) of sarcomas, including MFS, to identify immune-related biomarkers of prognostic and therapeutic relevance. Although data remain limited in MFS, existing evidence suggests a heterogeneous immune landscape, including: i) variable expression of immune checkpoint molecules such as Programmed Cell Death Protein 1 (PD-1) and Programmed Death-Ligand 1 (PD-L1), ii) presence of tumor-infiltrating lymphocytes, iii) alterations in antigen presentation pathways, and iv) a pronounced angiogenic signature. These findings underscore the potential role of immune biomarkers for patients’ clinical stratification and the consequent possibility of developing new immunotherapeutic strategies. This review will focus on the cellular and molecular architecture of immune infiltration, vascular remodeling, and lymphoid neogenesis, assessing their prognostic and predictive value as potential biomarkers. Finally, we will present ongoing clinical trials aimed at modulating the immune-vascular niche to inform innovative therapeutic strategies for this challenging sarcoma subtype.

## 1 Introduction

Myxofibrosarcoma (MFS) is a malignant soft tissue sarcoma (STS) that primarily affects the limbs of elderly patients, with a predilection for male subjects. It is characterized by myxoid stroma, pleomorphic tumor cells, and an infiltrative growth pattern. Although relatively rare, MFS poses significant clinical challenges due to its high propensity for local recurrence and metastasis. Understanding the pathobiology of MFS is crucial for developing effective treatment strategies, as its clinical behavior is strongly influenced by both intrinsic tumor cell properties and the surrounding tumor microenvironment (TME) (1).

The TME, consisting of various stromal cells, immune cell infiltrates, and extracellular matrix components, plays a crucial role in the progression, invasion, and resistance to therapy of tumors. In MFS, the interactions between malignant cells and the immune environment can influence key processes such as immune evasion and metastasis, ultimately impacting patient outcomes. Gaining a deeper understanding of these dynamic interactions may reveal novel therapeutic targets, particularly in the field of immunotherapy (2). Indeed, in recent years, immunotherapeutic strategies, including immune checkpoint inhibitors (ICIs), tumor vaccines, and adoptive cell therapies, have demonstrated efficacy in modulating the immune response against various types of tumors (3). However, the effectiveness of these therapies is often constrained by the complex, immunosuppressive microenvironment created by tumors like MFS. Consequently, innovative strategies targeting the tumor cells and the supporting microenvironment are crucial for improving therapeutic outcomes (4).

## 2 Clinical and pathological characteristics of myxofibrosarcoma

MFS originates from the connective tissue and is characterized by cellular pleomorphism, curvilinear vessels, myxoid stroma, and fibroblastic lesions (5). MFS represents approximately 5% of STS, which accounts for 1% of all tumors, making MFS a rare tumor. However, the increasing life expectancies in many Western countries suggest that it could become the most common STS in the future, considering its target population of elderly subjects (6).

Treatment options include surgery, radiotherapy, and chemotherapy. Surgery combined with neoadjuvant or adjuvant radiotherapy is the standard approach for localized disease. However, due to the MFS’s propensity to infiltrate the surrounding tissue, the tumor recurs in 50-60% of cases, and with every MFS recurrence, the metastatic potential increases. Chemotherapy remains the current standard for metastatic disease, despite its efficacy is still debated (7). The first-line treatment for recurrent or metastatic MFS is anthracycline, alone or combined with ifosfamide. Second-line chemotherapy treatments include high-dose ifosfamide, gemcitabine-based therapy, combined with docetaxel or dacarbazine, with encouraging responses (8).

Overall, despite multimodal treatment strategies, MFS remains a therapeutically challenging disease, marked by high local recurrence rates, limited systemic options, and an urgent need for more effective and tailored therapeutic approaches.

## 3 Histopathological features of myxofibrosarcoma

Gross examination of superficially located MFS generally shows a gelatinous to firm multinodular appearance. In contrast, deep-seated lesions often present as a single mass with deceptively infiltrative growth and frequent involvement of skeletal muscle. Areas of necrosis can be present in varying proportions. Histological presentation ranges from a broad spectrum of different degrees of aggressiveness, and the diagnosis hinges on the presence of invariable features, including multinodular growth with myxoid stroma, overt nuclear atypia, and delicate curvilinear blood vessels (**Table 1**) (1). Neoplastic cells are spindled to stellate, with slightly eosinophilic cytoplasm and hyperchromatic nuclei. Low-grade MFS harbors a lower density of cells, reveals mild nuclear atypia, and lies in an abundant myxoid stroma. Intermediate-grade lesions are characterized by increased cellularity, nuclear pleomorphism, necrosis, and mitotic activity (9, 10). High-grade MFS shares overlapping features with undifferentiated pleomorphic sarcoma (UPS), characterized by neoplastic cells exhibiting severe pleomorphism and bizarre, multinucleated cells, which grow in solid sheets and fascicles, lacking clear histological differentiation. Mitotic activity is typically brisk, and atypical mitoses are readily identifiable. In this context, the presence of myxoid stroma is crucial in distinguishing these two entities (11). Interestingly, the proportion of myxoid stroma and the number of curvilinear vessels had been respectively identified as prognostic factors in MFS. Indeed, higher amounts of myxoid stroma have been shown to correlate positively with better outcomes, whereas increased vascularity appears to predict a higher risk of metastasis (11, 12). Accordingly, Mentzel et al. highlighted a clear correlation between the intratumoral microvessel density and the histological progression of MFS (13). Indeed, neovascularization represents a crucial process for tumor growth, with significant consequences for clinical outcome. In this regard, vascular endothelial growth factor (VEGF) plays a key role in angiogenesis, and its expression in MFS has been well-documented; mRNA *in situ* hybridization revealed high expression of VEGF and thrombospondin-1 by MFS neoplastic cells (13), and immunohistochemistry (IHC) highlighted a diffuse and strong expression of VEGF-A and PDGFRα in MFS (14).

**Table 1:** Summary of morphological, immunohistochemical, and molecular features of myxofibrosarcoma according to the histological grade.

Grading*	Gross features	Histopathology		IHCPrognostic markers	Genetics	
Low grade	Superficial to deep-seated mass Gelatinous to solid appearance Ill-defined borders Variable necrosis	Multinodular growth Myxoid stroma Spindled to stellate fibroblastic/myofibroblastic cells with hyperchromatic nuclei Curvilinear blood vessels Infiltrative growth	Neoplastic cells set in abundant myxoid stroma Low to intermediate cellularity Mild to moderate nuclear atypia	CD34 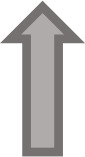 SMA	Gains of Ch7p, 7q, 9q, 12p, 12q, 17q, 19p, 19q, Xp, Xq Losses of Ch1q, 2q, 3p, 4q, 7p, 7q, 10p, 10q,11p, 11q, and 13q, 20p, and 21q High-level amplifications of Ch1, 5p, 20q Mutations in *TP53 CDKN1A, FGFR3, PTEN, RB1* CNA in *TP53, CDKN2A, CCND1, CCNE1, EGFR, EPHA3, EPHB1, FGFR1, JUN, NF1, RB1, RET* Other altered genes (including SNV, CNA, and fusions): *CDKN2B, KRAS, WNT11, NTRK1, MDM2, CDK6, GNAS, FOXA1, NKX2-1, SYK, JAK1, ATRX, TET2, MUC17, COL6A3, FLG, NLRP4, SLC12A5, VCAN, WDR87, ZNF680, ZNF780A*	Mixture of SNV, CNA and structural chromosomal abnormalities
				CD109 TEM1 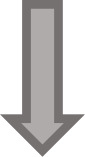 Ezrin MET		
High grade			Neoplastic cells arranged in sheets/fascicles with scant myxoid stroma Increased cellularity Severe nuclear atypia Pleomorphic multinucleated cells	CD34 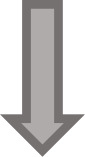 SMA		Increased cytogenetic alterations, with triploid and tetraploid karyotype Ch7 polysomy with overexpression of *MET* Amplifications in CCNE1, KIT, EGFR, RET, BRAF, NTRK2** Increased ‘SNV plus CNA’ in *TP53*
				CD109 TEM1 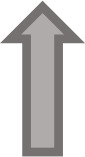 Ezrin MET		

Ch: chromosome; CNA: copy number alterations; IHC: immunohistochemistry; SMA: smooth muscle actin; SNV: single-nucleotide gene variants. *A two-tiered grading system has been used in the table for easier interpretation of the concepts. **These genetic alterations have been found in intra-tumoral high-grade areas within the same samples.

Although its diagnostic utility is limited, IHC has offered valuable insights into MFS TME and contributed to the identification of potential predictive biomarkers. For instance, the membrane-bound sialomucin-like endothelial ligand for the L-selectin molecule, CD34, predicts a worse overall survival (OS) when downregulated, as detected by IHC; indeed, high-grade MFS tends to lose CD34 (15). CD34 facilitates the endothelial adhesion of L-selectin–expressing lymphocytes, thereby influencing the organization of the TME (16). Stromal fibroblastic cells express CD34, contributing to fibrosis, tissue repair, and tumor stroma formation (15).

CD109 is a glycosylphosphatidylinositol-anchored protein that negatively regulates Transforming Growth Factor-β (TGF-β) signaling, thereby influencing cytokine secretion, immune cell recruitment, and macrophage polarization, with profound effects on the TME and cancer cell phenotype (17). TGF-β plays a pivotal role in regulating immune responses, cellular proliferation, differentiation, and extracellular matrix (ECM) production, exerting diverse, context-dependent effects in cancer biology (18). Notably, it shows an anti-oncogenic activity in the early phase of oncogenesis by inducing apoptosis and inhibiting cell cycle, whereas it promotes tumor progression and metastasis in advanced stages by fostering epithelial-mesenchymal transition and immune suppression (19). Although the mechanisms responsible for the dual role of TGF-β are not completely clear, p53 wild-type status seems to contribute to the early antitumoral activity, while its subsequent mutation unravels the pro-invasive and migratory effects of TGF-β in the late stage of the disease (18). Identified as a specific biomarker for MFS, CD109 expression carries adverse prognostic implications: CD109-positive cases show a 5-year OS of 0% compared with 77% in CD109-negative (20). Beyond its diagnostic and prognostic relevance, the inhibitory effect of CD109 on TGF-β signaling could mitigate tumor-promoting immunosuppression, restrain M2-like macrophage polarization, and restore T cell activity, thereby potentially enhancing anti-tumor immunity. However, the implications of CD109-mediated TGF-β inhibition on tumor progression in MFS have not yet been clearly investigated. Given its increased expression in high-grade MFS, CD109 has also been proposed as a potential therapeutic target (7).

Endosialin, also known as tumor endothelial marker 1 (TEM-1), is a type I transmembrane glycoprotein that belongs to the C-type lectin domain, with an active role in tumor-promoting mechanisms, favoring cell migration, as well as inducing an immunosuppressive TME (21), which makes it a valuable target for diagnostic and therapeutic strategies. In this regard, a diffuse and strong expression of TEM-1 was observed in 90–100% of MFS cases across two independent studies, each analyzing 33 samples, thereby supporting its potential as a biomarker for targeted approaches. These studies demonstrated that TEM-1 positivity in neoplastic cells was coupled with its absence in nearby healthy tissues, confirming that TEM-1 is a suitable marker for pre-operative tumor-targeted imaging of MFS (14, 22). Nonetheless, further investigations are warranted to validate TEM-1 as a reliable biomarker in MFS.

Ezrin is a member of the ezrin-radixin-moesin family, which drives multiple signaling programs related to substrate adhesion, cell survival, and migration. Ezrin has been identified as an independent prognostic factor for MFS disease-specific survival and metastasis-free survival. Moreover, its overexpression is associated with increased necrosis, mitotic activity, and histologic grade (23, 24). Ezrin also promotes macrophage polarization towards the pro-tumorigenic M2 subtype, favoring an immunosuppressive status of the TME, and fosters migration, invasion, and the production of pro-angiogenic factors (VEGF-A) and matrix metalloproteinase-9 (MMP-9) (24). Although the upstream mechanisms driving ezrin activation in MFS are not yet fully elucidated, preliminary studies suggest a potential involvement of c-MET. MET is a tyrosine-kinase receptor with high affinity for Hepatocyte Growth Factor (HGF), exerting crucial effects on the invasive growth program (25). MET seems to induce the N-terminal tyrosine phosphorylation of ezrin, which in turn affects cytoskeletal organization with subsequent morphogenetic modifications. Whether its upregulation is implicated in ezrin regulation, the role of MET is still to be elucidated (23). Intriguingly, IHC overexpression of MET has been observed in MFS with unfavorable features such as deep-seated location, high histological grade, large size, and advanced stage, with negative implications on OS and metastasis-free survival (26). These data raise interest in further exploring the role of MET as a possible predictive biomarker and tool for upcoming targeted strategies in patients with MFS.

## 4 The landscape of genetic aberrations in myxofibrosarcoma

MFS is one of the most complex types of sarcomas, characterized by a high degree of genomic instability due to the presence of a mixture of single-nucleotide gene variants (SNV), copy number alterations (CNA), and structural chromosomal abnormalities. The progression in MFS grade is also accompanied by an increase in cytogenetic alterations (27); indeed, triploid and tetraploid ranges are noted in the majority of high-grade MFS cases.

A comparative genomic hybridization (CGH) study showed gains of 19p and 19q, losses of 1q, 2q, 3p, 4q, 10q, 11q, and 13q, and high-level amplifications of the central region of chromosome 1, 5p, and 20q (28). In addition, gains of 7p21-22, 7q21-22, 7q31–35, 9q22, 12p13-pter, 12q15–21, 17q22–23, Xp11, and Xq12 and losses of 7p12, 7q11, 10p13–14, 10q25, 11p11–14, 11q23–25, 13q14–34, 20p11–12, and 21q22 were also documented (26, 29). It was suggested that polysomy of chromosome 7 might lead to the overexpression of the MET protein, whose high levels are associated with MFS deep location, higher grade, and more advanced stage (30). Recently, the FoundationOne^®^ Heme testing demonstrated the presence of an upregulation of HGF/MET signaling in a subset of MFS (31).

Although the mutational burden is lower than that of other types of cancer, Heitzer and colleagues, using a cancer hot spot panel, identified *TP53* mutations in 44% of MFS patients; in addition, variants in other genes, including *CDKN1A*, *FGFR3*, *PTEN*, and *RB1*, were observed, although at low frequency (*i.e.*, 1%) (32). In the same study, low-coverage whole genome sequencing (WGS) confirmed the variety of CNA from CGH studies, affecting known cancer driver genes, such as *CDKN2A*, *CCND1*, *CCNE1*, *EGFR*, *EPHA3*, *EPHB1*, *FGFR1*, *JUN*, *NF1*, *RB1*, *RET*, and *TP53*; interestingly, higher grade areas of the same tumor showed novel emerged focal amplifications including *CCNE1*, *KIT*, *EGFR*, *RET*, *BRAF*, *NTRK2* compared with the respective lower grade areas.

Whole exome sequencing (WES) of 41 paired MFS tumor and normal samples identified 127 recurrently mutated genes, but the only gene with mutations significantly associated with MFS was *TP53*. The recurrently mutated genes identified in this study included, besides *TP53* (22%), *NTRK1* (7%) and *NF1* (7%), but also previously unreported genes (*e.g.*, *ATRX*:10% and *TET2*: 7%) (33). By considering cumulative genetic alterations (*i.e.*, SNV, CNA, and fusion genes), a total of 14 genes were identified as significantly altered: *TP53*, *CDKN2B*, *CCND1*, *CDKN2A*, *KRAS*, *WNT11*, *NTRK1*, *MDM2*, *CDK6*, *GNAS*, *FOXA1*, *NKX2-1*, *SYK*, and *JAK1*. The same analysis on 116 MFS cases confirmed the presence of frequent driver mutations and CNA in *TP53* (46%), *RB1* (18%), *CDKN2A* (16%), *CDKN2B* (16%), *NF1* (11%), and *NTRK1* (9%). All these findings indicate central roles for dysregulation of p53 signaling and G1/S cell cycle in the development of MFS.

By integrating their findings with available TCGA datasets, Takeuchi et al. analyzed a total of 102 MFS samples. In addition to the genes previously identified (*i.e.*, *TP53*: 24.5%; *RB1*: 22.5%; *ATRX*: 14.7%; *NF1*: 8.9%; *TET2*:4.9%), several novel candidates for driver mutations were found including *MUC17* (24.5%), *COL6A3* (5.9%), *NLRP4* (5.9%), *SLC12A5* (5.9%), *VCAN* (5.9%), *WDR87*(5.9%) and *ZNF680* (4.9%). Beyond the well-characterized focal amplifications and copy number losses at known oncogene and tumor suppressor loci, they also identified novel genes in MFS affected by both mutations and CNAs. For example, alterations in the *FLG* gene, encoding the intermediate filament-associated protein filaggrin, and in ZNF780A, a zinc finger protein potentially involved in transcriptional regulation, were recurrently detected (34).

Recently, primary MFS cases were studied using IHC and genetic analysis (35), confirming that high-grade tumors were significantly associated with MET positivity. Moreover, in the group that underwent molecular screening, the presence of “SNV plus CNA” in *TP53* represents a risk factor for worse 5-year event-free survival. In conclusion, this study confirmed the high frequency of *TP53* alterations in MFS (86.4% of cases), highlighting their potential role in driving tumor aggressiveness and reinforcing the value of molecular profiling to better define the prognostic relevance of p53 in this malignancy.

Interestingly, beyond its classical cell-autonomous effects (i.e., induction of cell cycle arrest, apoptosis, or senescence, and promotion of DNA repair), p53 also plays a critical role in the TME by inhibiting angiogenesis and reducing the activity of immunosuppressive components, including regulatory T cells (Tregs) and myeloid-derived suppressor cells (MDSCs). In addition, p53 stimulates the expression of MHC class I molecules, upregulates the secretion of pro-inflammatory cytokines and immune-stimulatory molecules, and downregulates the inhibitory receptor Programmed Death-Ligand 1 (PD-L1). Through these mechanisms, p53 promotes the killing of cancer cells by cytotoxic T lymphocytes (CTLs) and natural killer (NK) cells, while also regulating macrophage polarization toward an antitumoral M1-like phenotype (36, 37). Consequently, alterations affecting the p53 protein can contribute to tumor immune evasion by reshaping the TME. Chen et al. explored the relationship between TP53 status (wild-type versus mutant) and the immune score in 254 sarcoma patients, including 25 (9.8%) cases of MFS, and found no significant correlation (38). It is important to note that TP53 mutations are functionally heterogeneous and can be classified into distinct functional categories (39). Therefore, current studies might be limited by a simplified patient stratification (e.g., wild-type vs. mutant). We can thus hypothesize that a comprehensive characterization of the p53 landscape in MFS and its correlation with the features of the TME may yield interesting results, as has been demonstrated in other cancer types (40, 41).

A summary of morphological, immunohistochemical, and molecular features of myxofibrosarcoma according to the histological grade are listed in **Table 1**.

## 5 Immunobiology of myxofibrosarcoma

A deeper understanding of the immunobiology of MFS is crucial for the development of targeted therapies capable of effectively harnessing the immune system against this rare and challenging malignancy.

### 5.1 Tumor-associated antigens

Cancer testis antigens (CTA) are expressed in the testes and various types of cancer but have limited expression in normal adult somatic cells and tissues. These antigens can be recognized by CTLs, making them an attractive target for antitumor immunotherapy (42). Among CTA, MAGE-A3 expression has been investigated in MFS as a tumor-associated antigen. Using the Cancer Genome Atlas and Cancer Cell Line Encyclopedia, and tissue microarray, Conley et al. showed that UPS and MFS express MAGE-A3, highlighting the possibility of its exploitation for immunotherapeutic approaches by adoptive T cell therapies (i.e., TCR-based immunotherapy targeting MAGE-A3) or vaccines. Indeed, MFS expresses Human Leukocyte Antigen (HLA)-class I molecules, and can therefore present antigens to lymphocytes in the immune infiltrate within TME (43). Also, other tumor-associated antigens, such as survivin and papillomavirus binding factor (PBF), together with HLA class I, were found to be expressed in MFS tissue by IHC analysis (44). IHC analysis detected New York esophageal squamous cell carcinoma-1 (NY-ESO-1) expression in a large cohort of sarcomas, including 17 MFS; interestingly, six out of 17 MFS showed positivity (45). Lastly, surface expression of Chondroitin sulfate proteoglycan 4 (CSPG4) was detected on a cohort of 100 MFS; higher CSPG4 expression was considered an unfavorable prognostic factor and correlated with an immune-excluded TME, making this a remarkable antigen for future targeted-cell-based immunotherapies (*i.e.*, CAR-T cells) (46).

Noteworthy, while in some cancers, *i.e.*, Non-Small Cell Lung Cancer (NSCLC), tumor cell PD-L1 positivity has been identified as a biomarker for ICI intervention (47), in MFS, it is still a matter of debate. Some authors indicate that PD-L1 expression is present in 15% of all sarcomas (34/222), with UPS having the highest prevalence, and MFS showing negative expression (48). Differently, in 16 cases of high-grade MFS, a high density of both CD8+ and FOXP3+ tumor-infiltrating lymphocytes was associated with PD-L1 positivity (49). Differences reported in the literature about PD-L1 expression may be due to the variety of PD-L1 antibodies used for IHC, differing cut-off values, and the evaluation methods for positivity.

### 5.2 Myxoid extracellular matrix

**Figure 1:**
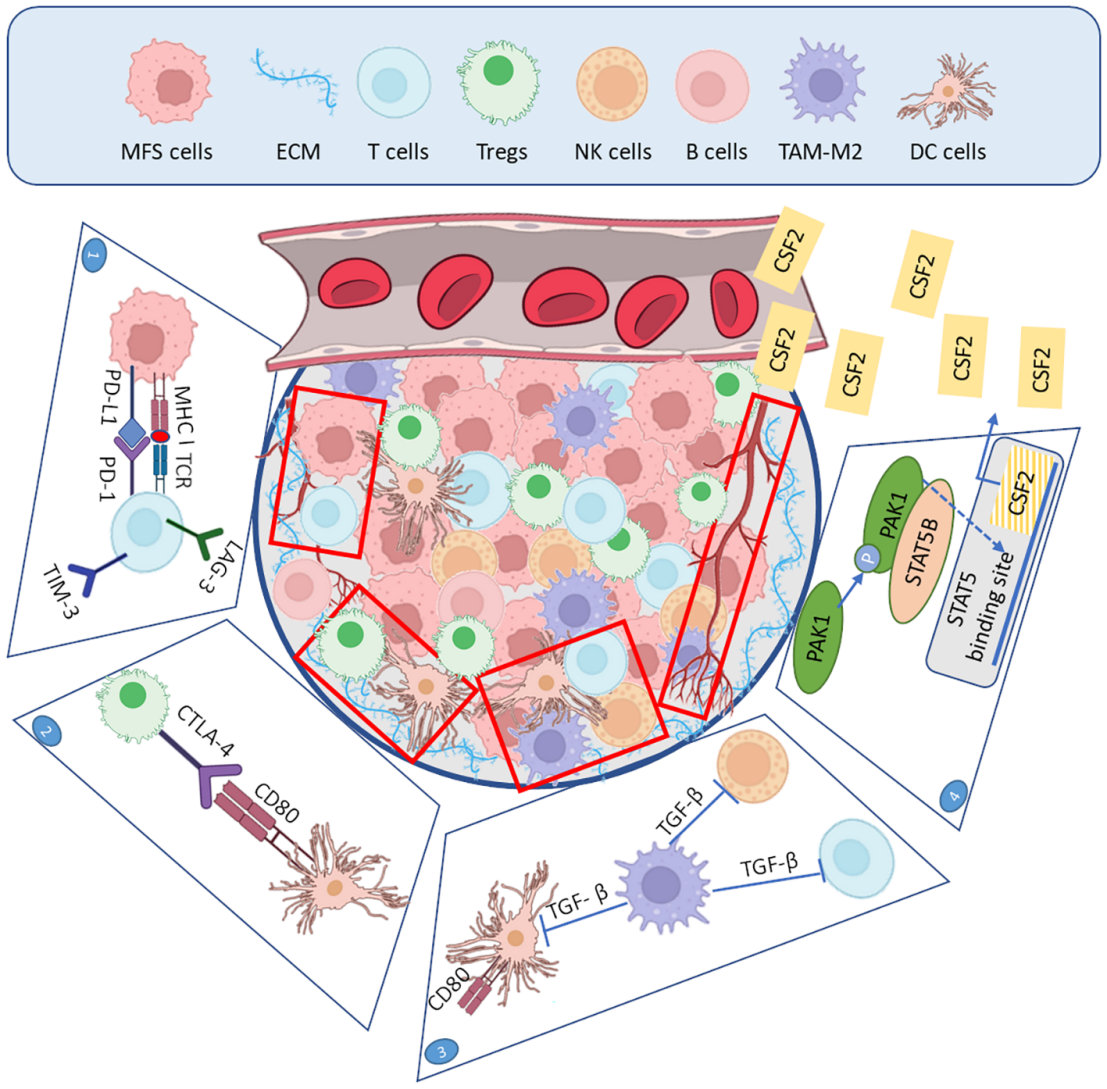
Composition of myxofibrosarcoma tumor microenvironment. Representation of key mechanisms that contribute to the establishment of an immunosuppressive TME, thereby potentially limiting responses to immunotherapies, as discussed in this review. Top panel (blue box): Cellular components of the MFS TME, including MFS cells, a dense ECM, T cells, Tregs, NK, B cells, TAM-M2, and DC. Box 1: Interaction between tumor and immune cells contributing to T cell dysfunction. Box 2: Tregs/DC interactions via the inhibitory receptor CTLA-4 binding to CD80, leading to reduced co-stimulatory signaling and impaired DC activation. Box 3: TAM-M2 releases TGF-β immunosuppressive cytokine, which inhibits DC maturation and suppresses both T cell and NK cell cytotoxic activity, thereby dampening antitumor immune responses. Box 4: MFS cells express PAK1 and promote angiogenesis through the STAT5B-mediated transactivation of CSF2, leading to increased CSF2/GM-CSF secretion. Elevated CSF2 levels promote tumor proliferation and angiogenesis.

MFS is characterized by a ECM where Glycosaminoglycans (GAGs) are the most important components and key players in tumor progression and cellular invasion (50). Among GAGs, hyaluronic acid (HA) contributes to a dense ECM, creating pressure on the surrounding area, which causes collapsing blood vessels, hypoxia, limited blood transport, and exhibits a more aggressive tumor phenotype (50). The myxoid ECM of MFS is heterogeneous and comprises also proteins, including albumin, that might play a role in the morphogenesis of this characteristic myxoid morphology (51) (**Figure 1**). Understanding the role of HA in MFS is crucial, as it offers a potential therapeutic target for intervention.

### 5.3 Tumor microenvironment

TME may influence response to immunotherapy due to the multiple interactions between cancer cells and immune cells, including Dendritic Cells (DC), tumor-infiltrating lymphocytes (TILs), and macrophages (47). A complete characterization of MFS’s TME is missing so far, but recent studies have started to characterize it, focusing on immune cell infiltration patterns (**Figure 1**).

In one study, integrating patients’ clinical data and genomic datasets, MFS samples were classified based on immune cell infiltration patterns and corresponding gene clusters. Algorithms allowed the authors to analyze and quantify immune cells on tumor tissue samples and stratify patients in the following clusters: i) with significantly high infiltration levels of CD4 T cells, activated NK cells, monocytes, M2 macrophages, ii) with B cells, resting NK cells, and M0 macrophages, and iii) with high CD8 T, follicular helper T, gamma delta T cells, and M1 macrophages. As expected, the last cluster, characterized by an immune-inflamed TME, was sensitive to immunotherapy (52).

#### 5.3.1 Dendritic cells

DC play a crucial role in antigen presentation, a process essential for initiating a strong and effective immune response against the tumor; their role in MFS has been rarely investigated, with the presence of DC and immature dendritic cells (iDC), as a favorable biomarker, having been associated with better disease-free survival in MFS (53). Soilleux et al. observed that MFS contained between 3% and 61% of DC expressing a C-type lectin, presumed to be infiltrating DC, regardless of tumor grade, and possibly involved in presenting tumor antigens to T lymphocytes (53).

#### 5.3.2 Tumor-infiltrating lymphocytes

TILs include various subtypes of lymphocytes, such as T (CD4+ and CD8+), NK, B, and Tregs, being the latter particularly associated with an increased risk of local recurrence, regardless of surgical margin status (54). Tregs are known to modulate the immune response and suppress the activity of other immune cells by multiple mechanisms, such as (i) cell-cell contact-dependent suppression through surface molecules such as Cytotoxic T-Lymphocyte Antigen 4 (CTLA-4), Lymphocyte Activation Gene 3 (LAG-3) and Programmed cell Death protein 1 (PD-1); (ii) downregulation of co-stimulatory molecules (CD80/CD86) on DC via CTLA-4; and (iii)secretion of immunosuppressive cytokine, notably TGF-β (55, 56). TGF-β not only enhances Treg function by inducing FOXP3 expression, but also decreases CD80/CD86 expression on antigen-presenting cells, particularly DC (56).

Smolle et al. demonstrated that high levels of Tregs in MFS are negative prognostic markers for local recurrence, indicating a role in immune evasion by dampening the antitumor immune response (54). Infiltrating T cells, expressing immune checkpoint molecules such as T-cell Immunoglobulin and Mucin-domain containing-3 (TIM-3) and LAG-3, have been identified in a significant proportion of MFS cases using tissue microarrays. Approximately 68% of MFS cases exhibited expression of LAG-3 and about 85% of TIM-3 (57), hypothesizing a role for these molecules in immune evasion, and the potential utility of ICIs targeting TIM-3 and LAG-3 as a promising treatment strategy. Moreover, higher expression levels of PD-L1, PD-1, TGF-β, and TIM-3 have been found in MFS compared to other sarcomas (58–60). These markers are often associated with immune modulation within the TME, suggesting that MFS has specific immune characteristics that may impact tumor progression and patient outcomes. Potential predictive biomarkers to ICIs (*i.e.*, pembrolizumab) include the presence of preexisting CD8+ T cells in the TME (60–62) or higher numbers of PD-1+ T cells at baseline and increased PD-L1 expression during treatment (49, 63).

#### 5.3.3 Macrophages

Macrophages represent an important component of the first-line defense against pathogens and tumor cells. Their presence in the TME has been strongly detected using the TCGA Project Management in UPS and MFS (58, 64). Indeed, tumor-associated macrophages (TAMs) are a major component of the immune infiltrate in MFS; Dancsok et al. demonstrated that MFS tumors are characterized by a higher proportion of M2-like (anti-inflammatory) macrophages compared to M1-like (pro-inflammatory) macrophages (65). This predominance of M2-like macrophages is associated with immunosuppressive functions that can promote tumor growth and metastasis. Computational analyses by CIBERSORT across sarcoma, including MFS, datasets have shown enriched presence of immunosuppressive myeloid populations, suggesting a TME supportive of MDSC infiltration (52, 60). However, direct studies on MDSC in MFS are absent.

Targeting the immunosuppressive components of the TME, *i.e.*, Tregs, MDSC, and TAM-M2, may represent a promising strategy to enhance the efficacy of immunotherapy in MFS.

### 5.4 Tertiary lymphoid structures

In recent years, the presence of Tertiary lymphoid structures (TLS), organized immune cell aggregates found in non-lymphoid tissues, including tumors, has been associated with improved patient outcomes and response to immunotherapy in various cancers (66). TLS in sarcomas correlates with improved outcomes and enhanced responsiveness to ICIs, particularly in UPS (67). In a large TCGA analysis of STS, Wang et al. applied a TLS gene signature and found that MFS and dedifferentiated liposarcoma had the highest TLS scores among STS histologies (68). These TLS in MFS patients could potentially influence prognosis and be a predictive biomarker of response to treatment, particularly immunotherapies. Indeed, in this context, intratumoral TLS can facilitate effective antitumor T-cell responses upon checkpoint inhibition.

### 5.5 Angiogenesis

Angiogenesis is the physiological process by which new blood vessels form from pre-existing vessels; tumors frequently stimulate angiogenesis to supply oxygen and nutrients. In MFS, a complex regulation of angiogenesis has been observed; indeed, an analysis of 43 MFS cases demonstrated that intratumoral microvessel density (assessed by CD31 staining) increases proportionally with histological grade, correlating with VEGF and with mRNA expression of its receptors, as well as with elevated levels of thrombospondin−1 and collagen I (13). Overexpression of p21-activated kinase 1 (PAK1) also contributes to pro-angiogenic phenotypes in MFS (69). Indeed, PAK1 promotes angiogenesis through the STAT5B-mediated transactivation of colony-stimulating factor 2 (CSF2, a.k.a GM-CSF), leading to increased CSF2 secretion. This pathway enhances tumor proliferation and angiogenesis, suggesting that targeting PAK1 could be a potential therapeutic strategy in MFS.

Understanding the immunobiology of MFS is essential for developing targeted therapies that can effectively harness the immune system to combat this challenging, rare malignancy.

## 6 Immune checkpoint inhibitors

The efficacy of immunotherapy by ICI relies on several factors, including the TME and the presence of specific genetic changes within tumor cells. The tumor mutational burden (TMB) that represent a measurement of how many genetic changes (*i.e.*, mutations) are found in the genome of tumor cells in a specific amount of DNA, is a predictive biomarker for immunotherapy-base responses; indeed, high TMB often correlates with increased neoantigen production, which can enhance immune system recognition of tumor cells, favoring response to ICIs (70). While skin melanoma or lung cancers show a very high TMB, MFS is characterized by a low TMB, making it challenging for immunotherapy treatments. PD-1 and CTLA-4 blockade revolutionized the treatment of melanoma and NSCLC, improving survival and offering durable responses (12–14) by reactivating the immune system’s ability to recognize and attack tumor cells (71). The most relevant benefit of ICIs in NSCLC was observed in those patients characterized by high PD-L1 expression (≥50%) on tumor cells (47) since it facilitates ICIs’ efficacy by increasing tumor visibility, and together with high TMB, provokes a stronger T cell-mediated immune response. This interplay underscores the potential of using PD-L1 expression and TMB as biomarkers to predict immunotherapy outcomes.

MFS may exhibit variable levels of immune infiltration, with some tumors expressing immune checkpoint molecules such as PD-L1. Those tumors with higher PD-L1 expression or a significant presence of TILs may respond better to ICIs (72, 73). For tumors resistant to ICIs, the strategy is to use a combination approach with chemo-, radio-, targeted- therapy that can efficiently circumvent tumor resistance to ICIs therapy (74). MFS response to immunotherapy is still under investigation, and so far, many studies have shown conflicting results of ICI as monotherapy or in combination (75).

In a multicenter phase 2 study (Alliance A091401, NCT02500797), nivolumab (*i.e.*, an anti-PD-1) alone or in combination with ipilimumab (*i.e.*, an anti-CTLA-4) was tested in sarcoma patients who had received at least one previous line of systemic therapy; in the combination therapy arm, the objective response rate (ORR) was 16%, and responses occurred in UPS, MFS, and leiomyosarcoma subtypes with manageable safety profiles of combined treatment, similar to other therapeutic interventions (76, 77). These results led to the planning of future phase III clinical trials, enriched for specific sarcoma subtypes with baseline TIL infiltration that are expected to show clinical efficacy to ICIs, including MFS. The combination of ipilimumab + nivolumab was used as a compassionate third-line therapy on a single patient with metastatic MFS. This treatment, followed by the administration of nivolumab alone, showed a partial response and stable disease without the onset of new lesions and with a manageable toxicity profile for 12 months (78). Similarly, a MFS patient resistant to standard therapy, despite negative PD-L1 status, had a complete response to ipilimumab + nivolumab treatment for over 3 years (79). Lastly, other two MFS patients have been described to receive benefit from immunotherapy-based treatments; a first patient with refractory metastatic MFS, resistant to conventional treatment, was treated with pembrolizumab (*i.e.*, and anti-PD-1), achieving partial response for 18 months (62), while a second patient diagnosed with a high-grade MFS and pulmonary metastases (progressed after surgery, chemoradiotherapy, and an angiogenesis inhibitor treatment) was treated with camrelizumab (*i.e.*, an anti-PD-1), showing metastases shrinkage and stabilization of disease for 18 months. This last result was attributed to high levels of PD-L1 expression in approximately 40–50% of tumor cells that, coupled with a higher TMB, likely contributed to enhanced immune responsiveness (61).

Responses to ICIs may also benefit from previous treatments with chemotherapy that generate high grades of mutations in tumors, creating an adequate scenario for ICI efficacy. Indeed, a temozolomide-resistant metastatic MFS patient, treated with seventh-line atezolizumab (*i.e.*, an anti-PD-L1) that showed elevated TMB, achieved a durable response (80). Preclinical and early clinical trials suggest that chemotherapy and radiation, by inducing immunogenic cell death, damage-associated molecular patterns (DAMPs), and inflammatory responses, can improve the efficacy of ICIs in sarcoma subtypes such as MFS, converting a “cold” into a “hot”, less immune suppressive TME, allowing for better immune activation (81, 82). Indeed, the randomized phase II trial SU2C SARC032 study (NCT03092323) investigated the addition of pembrolizumab to preoperative radiotherapy and surgery, showing a significant 15% improvement in disease-free survival with an Hazard Ratio (HR) of 0.61 (90% CI 0.39–0.96; p=0.035). In this trial, UPS, MFS, dedifferentiated, and pleomorphic liposarcoma were included, with UPS and MFS analyzed together if located deep to the fascia in muscle, being MFS about 10% in both arms. Although overall survival data are not statistically significant, probably because the study was underpowered for this endpoint, the trend favored the addition of immunotherapy. This study provides the first randomized evidence that immunotherapy can significantly enhance outcomes in localized high-risk extremity sarcomas (83). All these results highlight that PD-L1 expression and TMB can be modified after standard therapy courses, particularly in MFS progressing after chemotherapy, making ICIs treatment more effective.

## 7 Immunotherapeutic approaches targeting the tumor microenvironment

Immunotherapy targeting the TME has emerged as a promising approach to cancer treatment. The possibility of targeting the extracellular matrix, immune cell composition, angiogenesis, favoring immune cell infiltration, or exploring alternative immune checkpoints may serve as a viable strategy to potentiate antitumor response alongside ICI. For instance, the dense, myxoid MFS ECM is mainly composed of HA, and targeting HA may be considered an interesting approach to favor anti-tumor therapy delivery. This strategy has been explored in other cancers, resulting in the reduction of interstitial pressure, improvement of vascular function, and enhanced efficacy of treatments (50). The expression of tumor-associated antigens, such as survivin and PBF, in a patient with MFS positive for HLA class-I, was exploited by peptide vaccination therapy using PBF and survivin peptides in incomplete Freund’s adjuvant, and PEG-conjugated interferon-α. The patient showed a strong response to the peptide vaccinations, which was increased after the administration of nivolumab (44). Increased knowledge of MFS TME composition, *i.e.*, Treg, MDSC, and TAM, demonstrates that an immunosuppressive environment will poorly respond to ICIs (47), thus, favoring immune evasion.

### 7.1 Macrophage targeting

#### 7.1.1 CD47-Signal regulatory protein α axis

Approaches targeting macrophages are being developed, and particularly interesting is the CD47-Signal regulatory protein α (SIRPα) axis, a ‘macrophage immune checkpoint’ (84). When CD47 binds to SIRPα, the “don’t eat me” signal is activated, inhibiting macrophage-mediated phagocytosis (85). Anti-CD47 antibodies re-enable macrophage-mediated phagocytosis of tumor cells, inducing M1 polarization (86). MFS is highly positive for CD47, particularly in the highest grades, although it is not a prognostic factor of OS (87). CD47-blocking therapies showed preclinical effectiveness in promoting phagocytosis across various tumor types and produced an antitumor cytotoxic T-cell immune response (88, 89). Targeting CD47 in combination with an ICI reactivates TAM-M1 and T cell functions, possibly generating tumor growth reduction. Simultaneous blockade of PD-1/PD-L1 and CD47/SIRPα by a CD47/PD-L1 bispecific antibody induced robust macrophage-mediated phagocytosis of tumor cells and demonstrated potent therapeutic efficacy across three distinct mouse models, supporting its rationale for applications in PD-L1 and CD47 double-positive cancers (90, 91). Unfortunately, adverse effects need to be considered since CD47 expression is found in normal cells, and its targeting may lead to off-target effects like anemia, thrombocytopenia, and leukopenia (92). Clinical targeting with a soluble recombinant fusion protein targeting CD47 has been exploited in a phase I clinical study (NCT02890368, **Table 2**) where a dose escalation trial of intratumoral injections of TTI-621 (SIRPα-IgG1 Fc) was applied to subjects with relapsed and refractory percutaneously-accessible solid tumors, including STS, alone or in combination with: ICI (*i.e.*, anti-PD-1/PD-L1), pegylated interferon-α2a, talimogene laherparepvec, or radiation. This trial terminated in 2020, and only results for mycosis fungoides or Sézary syndrome were published (93), but no data were reported for the STS included in the study.

**Table 2:** Concluded clinical trials involving MFS patients in immunotherapy regimens primarily focused on ICIs (clinicaltrials.gov). Last accessed October 2025.

NCT	Trial name	Treatment arms	Biological target	Phase
NCT02500797	Randomized Phase II Study of Nivolumab With or Without Ipilimumab in Patients With Metastatic or Unresectable Sarcoma	- Arm I: nivolumab intravenous (IV) - Arm II: nivolumab IV + ipilimumab IV	PD-1 CTLA-4	II
NCT03092323	SU2C-SARC032: A Phase II Randomized Controlled Trial of Neoadjuvant Pembrolizumab With Radiotherapy and Adjuvant Pembrolizumab in Patients With High-Risk, Localized Soft Tissue Sarcoma of the Extremity	Neoadjuvant pembrolizumab with concurrent radiotherapy, followed by surgical resection and adjuvant pembrolizumab	PD-1	II
NCT02890368	A Phase 1 Dose Escalation Trial of Intratumoral Injections of TTI-621 in Subjects With Relapsed and Refractory Percutaneously-Accessible Solid Tumors and Mycosis Fungoides	- TTI-621 Monotherapy Escalation - TTI-621 Monotherapy (Single Lesion) - TTI-621 Monotherapy (Multiple Lesions) - TTI-621 + PD-1/PD-L1 Inhibitor - TTI-621 + Pegylated Interferon-α2a - TTI-621 + T-Vec - TTI-621 + Radiation	CD47 PD-1 PD-L1	I
NCT04242238	A Phase 1b Dose Escalation and Dose Expansion Study of a CSF1R Inhibitor (DCC-3014) Administered Concurrently With an Anti-PD-L1 Antibody (Avelumab) in Patients With Advanced High-grade Sarcoma	DCC-3014 by pill + Avelumab IV	CSF1R PD-L1	Ib
NCT02575066	Phase II Clinical Study of Concurrent Pazopanib for Non-metastatic Sarcoma Patients to be Treated With Radiotherapy, Localized in the Extremities, Trunk and Chest Wall or the Head and Neck Region (PASART-2)	Radiotherapy combined with pazopanib	VEGFR	II

#### 7.1.2 Colony-stimulating factor 1 receptor pathway

Another target for TAMs is the Colony-Stimulating Factor 1 Receptor (CSF1R) pathway, which is critical for the survival and maintenance of the pro-tumoral M2 TAM subtype. Blocking CSF1R can reduce M2 populations and reprogram the TME towards anti-tumoral activity, not only disrupting the recruitment and survival of immunosuppressive M2-like TAM, but also of MDSC (94). Preclinical studies, in a glioma model, showed that CSF1R inhibition can synergize with anti-PD-1 antibodies, improving their efficacy by reducing TAM-mediated immune suppression and enhancing T-cell infiltration *in vitro* and *in vivo* (95). Specifically, the CSF1R inhibitor pexidartinib suppresses survival, migration, and M2 polarization of sarcoma TAMs *in vitro* and significantly inhibits osteosarcoma growth and metastatic spread *in vivo*, in a syngeneic murine model (96). Moreover, the combination of pexidartinib with sirolimus (*i.e.*, mTOR inhibitor) resulted in tumor growth suppression and a deep decrease in TAMs compared with mice treated with pexidartinib alone (97); this combination was demonstrated to be safe (98). Lastly, phase I/II trials are assessing the safety and efficacy of CSF1R inhibitors (*i.e.*, BLZ945) in combination with spartalizumab (*i.e.*, an anti-PD-1) in advanced/metastatic solid tumors (NCT02829723) without specific information on the number of STS included in the trial. An interventional phase Ib study (NCT04242238, **Table 2**) evaluated the concurrent administration of DCC-3014 (*i.e.*, CSF1R inhibitor) and avelumab (*i.e.*, anti–PD-1 antibody) in patients with advanced high-grade sarcoma, to deplete immunosuppressive M2 macrophages. The regimen was safe and tolerable, and in select patients, it reduced circulating MDSC and Treg while increasing tumor-infiltrating T cells. However, despite encouraging preclinical data, the clinical activity was limited, with no objective responses observed and a median progression-free survival (mPFS) of 1.55 months (95% CI, 1.25–1.78) (99). The discrepancy between the promising preclinical results of macrophage-targeting strategies and their limited clinical efficacy may be attributed to multiple, yet poorly understood factors, most likely related to the complex role of TAM activity within the broader TME and its systemic interactions. This highlights once again the critical need for more reliable preclinical models to better predict clinical outcomes.

#### 7.1.3 Prostaglandins

Prostaglandins (PGs) are different lipid mediators derived from arachidonic acid that, interacting with specific G-protein-coupled receptors (GPCRs), play a crucial role in inflammatory responses. Among PGs, PGD2 is produced by activated mast cells, macrophages, and T helper (Th) 2 cells and binds to a D-type prostanoid receptor (DP or DP1), inhibiting NK cytotoxic activity, and Th1 and DC differentiation, while PGE2, produced by epithelial cells, fibroblasts, and infiltrating inflammatory cells, exerts its effects through four GPCRs (EP1, EP2, EP3, and EP4), impairing NK functions, T cell receptor (TCR) signaling, and facilitating TAM polarization towards the M2 (100). Targeting PG receptors is pursued in an ongoing clinical trial as a single drug or in combination with ICIs (INVOKE trial, NCT06789172, **Table 3**, see section 8 Ongoing Clinical Trials).

**Table 3:** Ongoing clinical trials involving MFS patients in immunotherapy regimens that primarily focused on ICIs (clinicaltrials.gov). Last accessed October 2025.

NCT	Trial name	Treatment arms	Biological target	Phase
NCT04480502	ENVASARC: Envafolimab and Envafolimab with Ipilimumab in patients with Undifferentiated Pleomorphic Sarcoma or Myxofibrosarcoma (ENVASARC)	- Cohort A/C: Envafolimab alone- -Cohort B/D: Envafolimab + Ipilimumab	PD-L1 CTLA-4	I/II (pivotal)
NCT03425279	A Phase 1/2 dose escalation and dose expansion study of Mecbotamab Vedotin (BA3011) alone and in combination with Nivolumab in adult and adolescent patients 12 years and older with advanced solid tumors	- Phase1: dose escalation (BA3011 ± Nivo) - Phase2: expansion in UPS/MFS with BA3011 ± Nivolumab	AXL PD-1	I/II
NCT04332874	A study of Pembrolizumab plus local chemotherapy using Isolated Limb Infusion (ILI) for patients with sarcoma in the arm or leg	ILI (melphalan + dactinomycin) + Pembrolizumab every 21 days	DNA and gene transcription PD-1	II
NCT04420975	Nivolumab and BO-112 before surgery for the treatment of resectable soft tissue sarcoma	BO-112 intratumorally + Nivolumab IV + RT	PD-1 TLR3 RIG-I MDA-5	II
NCT06789172	A Phase 1, first-in-human study of OKN4395 and Pembrolizumab in patients with solid tumors (INVOKE)	- Phase 1a: dose escalation OKN4395 ± Pembro -Phase 1b sarcoma cohort: OKN4395 with or without fasting	PG receptors (EP2, EP4, DP1) PD-1	I

#### 7.1.4 Indole 2,3 dioxygenase 1

Indoleamine 2,3-dioxygenase (IDO) 1 is an intracellular enzyme expressed by tumor, endothelial, and DC and macrophages that depletes tryptophan, thus inhibiting T cell functions (101). In preclinical models, the combination of the IDO inhibitor epacadostat and anti-PD-1/PD-L1 or anti-CTLA-4 antibodies suppressed tumor growth more effectively than the drug alone through reactivation of anti-cancer immunity (102). MFS specimens showed positivity for IDO and kynurenine (103). A phase 2 study that evaluated epacadostat and pembrolizumab in patients with advanced sarcoma, including 2 MFS, demonstrated that the combination was well tolerated but with limited antitumor activity (103). The study did not specify how MFS responded to combination therapy.

### 7.2 Targeting of angiogenetic pathways

VEGF plays a prominent role in angiogenesis (104) and is highly expressed in MFS (13, 22), making it a possible target for therapy. Bevacizumab is the first anti-angiogenic drug approved by the U.S. Food and Drug Administration (FDA) for use in combination with chemotherapy for metastatic colorectal cancer (105). Targeting VEGF receptors (VEGFR) using small-molecule receptor tyrosine kinase inhibitors (RTKIs), such as pazopanib, has also been an important anti-angiogenic strategy (104). An Indian retrospective study evaluating 33 cases of rare subtypes of unresectable/metastatic STS, 4 of which were MFS, who received pazopanib, showed that this drug was active and achieved 50% stabilization and 50% progression of disease (106). In a retrospective study, out of eight MFS patients treated with pazopanib, six had stable disease, two for a long time, and two had progressive disease (107). Neo-adjuvant pazopanib and the concurrent external beam radiotherapy for high-risk, localized STS, including 8 MFS, exhibited, for one patient, a radiological partial response and, for the remaining patients, a stabilization of the disease [NCT02575066, **Table 2** (108)]. The combination of pembrolizumab and pazopanib is under investigation in patients with advanced renal cell carcinoma (109). The possibility of applying this combination treatment in MFS is an emerging area of interest since VEGFR-targeting may modify the TME, potentially increasing immune cell infiltration and making tumors more “visible” to the immune system, improving immune-mediated tumor destruction. Indeed, a UPS patient who progressed after two lines of palliative combination chemotherapy had remarkable radiological and clinical improvement after treatment with pazopanib in combination with pembrolizumab (110). Studies on MFS patients are still missing.

### 7.3 Alternative approaches

Direct targeting of tumor cells may constitute an alternative approach to be coupled with ICIs, and oncolytic viruses may be exploited to replicate within and lyse tumor cells, releasing tumor antigens and promoting regional and systemic antitumor immunity. Indeed, a study combining pembrolizumab with talimogene laherparepvec (*i.e.*, T-VEC), a modified herpes simplex virus-1 oncolytic virus (111), demonstrated manageable safety and antitumor activity in sarcomas, including MFS. This study paved the way for the usage of this combination in a neoadjuvant setting for localized and operable UPS and MFS (112).

## 8 Ongoing clinical trials

Evidence supporting the role of immunotherapy in MFS remains scarce, as prospective clinical trials have predominantly included MFS within heterogeneous STS cohorts rather than evaluating it as a distinct histological entity. Moreover, sub-analyses from these studies, even in selected ‘immune-sensitive’ sarcoma histotypes such as MFS, have underscored the need to modulate the tumor microenvironment to potentiate the immune response elicited by conventional immunotherapy agents. Therefore, several ongoing and recently completed studies are providing important insights into the modulation of the TME in MFS (**Tables 2**, **3**).

The ENVASARC trial (NCT04480502, **Table 3**) represents a pivotal study specifically designed to include patients with UPS and MFS in dedicated cohorts and evaluate subcutaneous administrations of Envafolimab (*i.e.*, anti-PD-L1) alone or in combination with ipilimumab. The primary objective of each parallel cohort is to demonstrate an ORR, while secondary endpoints include duration of response, PFS, and OS (113). Unfortunately, results did not meet the primary endpoint (ORR<11%).

In the BA3011 study (NCT03425279, AXL-targeted antibody-drug conjugate (ADC), mecbotamab vedotin, is tested alone and with nivolumab in patients with advanced sarcomas, including MFS. **Table 3**). The rationale for combining this ADC with nivolumab is intriguing, as AXL overexpression is associated with aggressive behavior and immune resistance in sarcomas. In fact, preliminary published results from the first part of the study revealed a PFS of 39.9% and a disease control rate of 41.1%, with 5 responding patients (114).

A different strategy is employed in the phase II study of pembrolizumab combined with isolated limb infusion (NCT04332874, **Table 3**). By delivering high concentrations of melphalan and dactinomycin, which target cancer cell DNA and gene transcription, it is expected to induce antigen spread and enhance local tumor immunogenicity, potentiating systemic immune responses (115). This approach may be particularly relevant for extremity MFS, where repeated local recurrences are a major clinical challenge.

A triplet strategy is under investigation in the BO-112 neoadjuvant trial (NCT04420975, **Table 3**) that integrates systemic nivolumab, intratumoral administration of BO-112, and radiotherapy before surgery. BO-112 is a nanoplexed poly I:C polymer that mimics viral dsRNA functioning as a pathogen-associated molecular patterns (PAMPs), triggering the immune system as if a viral infection were present. It activates key innate immune receptors Toll-like receptor 3 (TLR3), retinoic acid-inducible gene-I (RIG-I), and melanoma differentiation-associated protein 5 (MDA-5) (116). BO-112 showed therapeutic efficacy if given intratumorally in transplantable mouse tumor models, via activation of conventional DC (117). Preclinical studies demonstrated that BO-112 synergizes with radiotherapy and elicits CD8 antitumor immune responses (118). This approach once again seeks to combine different treatment modalities to increase tumor immunogenicity and amplify the systemic antitumor immunity elicited by ICIs (119).

The INVOKE trial (NCT06789172, **Table 3**) investigates the novel immunomodulator OKN4395 (triple inhibitor of PG receptors) alone and in combination with pembrolizumab in patients with advanced solid tumors, including MFS. Of particular interest, in the sarcoma-specific cohort of the expansion phase, this drug is being tested as monotherapy in combination with fasting as a metabolic intervention, based on the growing recognition that tumor metabolism and nutrient availability can profoundly shape the TME. This trial is unique compared with other ongoing studies and could potentially influence future treatment strategies, although its design as a ‘multiple sarcoma histology’ trial remains a well-recognized limitation.

Altogether, these trials illustrate the wide spectrum of multimodal approaches being explored in MFS immunotherapy, combining traditional strategies with innovative modalities targeting the TME, including ADC, radiotherapy, metabolic interventions, macrophage-directed agents, and intratumoral immunostimulants. While some encouraging signals are emerging, definitive evidence is still lacking. Most of the ongoing clinical trials have exploratory objectives, such as studying the composition of TME (*i.e.*, T cell infiltration), defining immunologic events that correlate with clinical outcomes during treatment, and investigating the mechanisms responsible for enhanced T cell infiltration into tumors following treatment. Results are expected to expand the knowledge of MFS TME and how to refine it to increase responses to immunotherapy.

## 9 Discussion and future perspectives

Considering the controversial data in the literature on MFS responsiveness to ICI, future clinical trials exploring combination strategies with chemo- and radio-therapy are mandatory. Furthermore, studies on adequately sized cohorts of MFS patients are needed to clarify whether these tumors should be classified as immunologically “hot” or “cold”. To date, only a few investigations have addressed this issue, and their conflicting results generally suggest that MFS represents a “cold” tumor type characterized by an immunosuppressive microenvironment and low mutational burden. However, complementary treatments, such as chemo- and/or radio-therapy, possibly generate high grades of mutations in tumors, thus converting an immunologically “cold” TME into a “hot,” more immune-permissive state, and generating an adequate scenario for ICI efficacy. Indeed, this happened in the SU2C SARC032 study, where pembrolizumab was combined with preoperative radiotherapy and surgery.

Unfortunately, studies on MFS are limited by the small number of patients enrolled, reflecting the rarity of the tumor, and by the average age of patients, who are frequently ineligible for trials due to health-related exclusions.

A deeper analysis of the MFS TME, focusing on immune checkpoints and immune cell populations, using advanced techniques, *i.e.*, multiplex immunofluorescence and multi-omics, is essential to shed light on tumor-immune interactions and identify biomarkers that may define a subset of MFS patients that may benefit from immunotherapy. Notably, a recently published study showed that approximately 68% of MFS express LAG-3, and about 85% TIM-3 (60). TIM-3 and LAG-3 are alternative immune checkpoints whose targeting by specific antibodies is currently under investigation in clinical trials in advanced solid cancers (120, 121), opening the way for similar investigations in MFS.

The possibility of testing immunotherapeutic combinations based on both conventional and new ICIs must be pursued in preclinical studies. Moreover, 2D or 3D patient-derived primary cell lines, as well as tumor specimens’ growth as organoids, can be exploited for immune-based combinations in an autologous experimental setting. It is important to emphasize that MFS tumors display substantial genetic and histological heterogeneity, making it challenging to develop models that faithfully recapitulate all aspects of the disease (33). Also, replicating the complexity of interactions of tumor cells with immune cells, ECM, and blood may be difficult, even in 3D or organoids (6). Then new and more sophisticated models or culture conditions are needed.

Alternatively, immunocompetent murine models with mouse tumors implanted in syngeneic mice would be the best setting to test new therapies; unfortunately, such models are not yet available for MFS, and the use of humanized mouse models bearing patient-derived xenografts still has some limitations, such as the development of graft versus host disease across all.

Future efforts should prioritize research on this rare tumor by developing models to better understand MFS pathophysiology and enhance treatment strategies. A multidisciplinary approach, bringing together oncology, immunology, bioengineering, and computational modeling, is essential to gain deeper insights and ultimately improve patient outcomes.
